# CLSPCOL rescues Alzheimer’s disease mouse models

**DOI:** 10.1515/tnsci-2022-0209

**Published:** 2022-02-02

**Authors:** Shinya Kusakari, Mikiro Nawa, Yuichi Hashimoto, Masaaki Matsuoka

**Affiliations:** Department of Pharmacology, Tokyo Medical University, 6-1-1 Shinjuku, Shinjuku-Ku, Tokyo 160-8402, Japan

**Keywords:** Alzheimer’s disease, calmodulin-like skin protein, adiponectin

## Abstract

Calmodulin-like skin protein (CLSP) inhibits Alzheimer’s disease (AD)-related neurotoxicity. The activity of CLSP is reduced in AD. To restore the CLSP activity, we developed a hybrid peptide named CLSPCOL, consisting of CLSP(1–61) and the collagen-homologous region (COL) of adiponectin. It was previously shown that the CLSPCOL-mediated restoration of the reduced CLSP activity alleviated memory impairment and neuronal synaptic loss in *APPswe/PS1dE9* double transgenic mice (APP/PS1 mice) at an advanced phase. Here, we examined whether CLSPCOL is effective against the memory impairment of the APP/PS1 mice at an early phase, and the memory impairment, caused by the temporal disturbance of the cholinergic neurotransmission, that mimics a part of AD-linked neuronal abnormality. The CLSPCOL-mediated restoration of the CLSP activity corrected the impairment in acquisition of fear-conditioned memory at an early-phase AD model. A single subcutaneous injection of CLSPCOL rescued the short-term working memory impairment, caused by subcutaneous injection of scopolamine. We have concluded that CLSPCOL is a promising disease-modifying therapeutic agent for not only the advanced phase but also the early-phase AD. It also serves as a symptomatic modifier of AD by potentiating the cholinergic neurotransmission.

## Abbreviations

AD: Alzheimer’s disease
APP: amyloid β precursor protein
APP/PS1 mice: *APPswe/PS1dE9* double transgenic mice
CLSP: calmodulin-like skin protein
CLSP(1–61): the N-terminal 1-61 amino acid region of CLSP
CNS: central nervous system
COL: collagen-homologous region
CSF: cerebrospinal fluid
CLSPCOL: a hybrid protein of CLSP(1–61) and the collagen-homologous region of adiponectin
htHNR: heterotrimeric humanin receptor
ISF: interstitial fluid of brain
PS1: presenilin 1
SA: spontaneous alternation
WT: wild type

## 1 Introduction

Humanin and calmodulin-like skin protein (CLSP) are physiological agonists of the heterotrimeric humanin receptor (htHNR), which consists of ciliary neurotrophic factor receptor α, WSX-1, and gp130 [1,2,3]. They directly inhibit Alzheimer’s disease (AD)-related neurotoxicity *in vitro* and *in vivo* and keep neurons fully intact in an amyloid β-independent fashion [1,2,4]. *In vivo* humanin concentrations are too low for humanin to show agonistic activity via the htHNR [5], whereas the concentrations of CLSP in the sera and the central nervous system (CNS) are sufficient [6]. Thus, it is highly likely that CLSP, rather than humanin, plays a central role in the activation of the htHNR *in vivo* [3].

In the human cerebrospinal fluid (CSF), the concentration of CLSP is 3–6 nM [6], which is more than the 50% effective concentration of CLSP (100 pM) [2,7], and concentrations of apolipoprotein E, a CLSP inhibitor, range from 40 to 200 nM [8,9,10]. Furthermore, there are some other CLSP inhibitors, such as calreticulin and 14-3-3 proteins [7], whose concentrations in the CNS are unknown. In the presence of various CLSP inhibitors whose concentrations are much higher than those of CLSP, the activity of CLSP is predominantly protected by adiponectin [7]. Adiponectin binds to the humanin-homologous region of CLSP whereas the CLSP inhibitors such as apolipoprotein E bind to the C-terminal region of CLSP, which is outside the humanin-homologous region [7]. In AD, however, the adiponectin concentrations are downregulated and CLSP activity appears insufficient to counteract AD-linked neurotoxicity [7]. The insufficiency in the CLSP activity likely results in the appearance of neurotoxicity in AD.

To restore the reduced CLSP activity efficiently, we developed a hybrid peptide named CLSPCOL, which is a hybrid peptide consisting of CLSP(1–61) and the collagen-homologous region (COL) of adiponectin [7]. CLSP(1–61) is fully active in inhibiting AD-related neurotoxicity and contains the blood–brain barrier-penetrating domain [7]. However, it is deficient in the binding sites of CLSP inhibitors [7]. The COL of adiponectin is the minimum required region of adiponectin that protects and potentiates CLSP activity by binding to the humanin-homologous domain of CLSP [7].

We previously showed that the CLSPCOL-mediated restoration of the CLSP activity alleviated memory impairment, using a modified Morris water maze test, and improved neuronal synaptic loss, in *APPswe/PS1dE9* double transgenic mice (APP/PS1 mice), at an advanced phase [7]. However, it remains unknown whether CLSPCOL is effective against the memory impairment in AD models at an early phase of memory impairment. It was also previously shown that humanin or CLSP improved the cholinergic neurotransmission [11,12]. It remains unknown whether the CLSPCOL is also effective against memory impairment, caused by the temporal disturbance of the cholinergic neurotransmission, that mimics a part of AD-linked neuronal abnormality.

In the current study, we examined the anti-AD effect of CLSPCOL in the APP/PS1 mice at a younger age (early-phase AD mouse model), using another memory test, the fear conditioning memory test. We also performed a Y-maze test to show that a single subcutaneous injection of 5 nanomol of CLSPCOL rescues memory impairment caused by scopolamine, an M2 muscarinic receptor antagonist.

## 2 Materials and methods

### 2.1 Mouse CLSPCOL

The amino acid sequence of the mouse CLSPCOL (mCLSPCOL: MSHGFTKEEVAEFQAAFNRFDKNKDGHISVEELGDVMKQLGKNLPEKDLKALISKLDTDGDGHPGHNGTPGRDGRDGTPGEKGEKGDAGLLGPKGETGDVGMTGAEGPRGFPGTPGRKGEP) is composed of the N-terminal 61 amino acids of the mouse CLSP-1, CLSP(1–61), and the 60-amino-acid-long collagen-homologous region (48–107 amino acids) of the mouse adiponectin that is directly fused. The mouse CLSPCOL was chemically synthesized by BioSynthesis (Lewisville, TX, USA). Its purity was more than 85%. Mass spectrometry analysis using MALDI-TOF was performed to confirm the molecular weight of the product. It was dissolved in sterile Milli-Q water (Millipore, Bedford, MA, USA) for use.

### 2.2 Mouse handling and the administration of scopolamine and CLSP for memory behavior tests

All mice used in the current study were male. The number of mice and the age of mice used in the experiment are presented in each figure legend.

APP/PS1 mice (B6.Cg-Tg(APPswe,PSEN1dE9)85Dbo/Mmjax, Cat# 005864, RRID:MMRRC_037568-JAX) were obtained from the Jackson Laboratory (Bar Harbor, ME, USA). They were bred and maintained at the Pre-clinical Research Center of Tokyo Medical University under specific pathogen-free conditions. They were housed in an air-conditioned room with a 12/12-h dark/light cycle and the start of light cycle at 8:00 am. After weaning at 1 month of age, the mice were genotyped using tail specimens and identified using ear-punched IDs. Only male mice were used for the experiments. Six mice of the same genotype were bred in cages. The same reagents were administered to all mice in each cage. The experimenters were not blinded to the genotypes of mice which they were handling. Mouse CLSPCOL in saline or saline without CLSPCOL was subcutaneously injected into the male APP/PS1 mice aged 12 months. As a control group, saline without CLSPCOL was subcutaneously injected into the male littermate wild-type (WT) mice aged 12 months. Five nanomol of mCLSPCOL were injected every other day for the first 13 days and every day for the last 16 days. The schedule for the subcutaneous injection of mouse CLSPCOL is illustrated in Figure S1.

For the treatment with scopolamine hydrobromide, male Jcl:ICR mice aged 10 weeks were purchased from Japan SLC Inc. (Shizuoka, Japan). Scopolamine hydrobromide (Cat. # S0929) was purchased from Sigma-Aldrich (St. Louis, MO, USA). Scopolamine (1 mg/kg mouse) with 5 nanomol of CLSPCOL or saline was subcutaneously injected into each mouse at 1 h before the Y-maze test.


**Ethical approval:** The research related to animals’ use has been complied with all the relevant national regulations and institutional policies for the care and use of animals. All animal experiments were approved by the Institutional Animal Care and Use Committee of Tokyo Medical University (No. H290026) and were conducted according to the Society’s Policies on the Use of Animals in Neuroscience Research.

### 2.3 A schedule of memory tests for APP/PS1 mice

Sample sizes were determined according to the previous study (Figure S1) [7]. Male APP/PS1 mice and their littermate WT mice at an age of 12 months were automatically allocated to groups by their identification numbers that were assigned when their genotype was determined. Experimenters were not blinded to mouse groups because they are involved in the handling of all breeding steps. The mice were subjected to the forward training session of the modified Morris water maze test on the 11th–13th days and the reverse training session on the 17th and 18th days [7]. After the water maze test was finished, spatial working memory performance was evaluated by monitoring spontaneous alternation (SA) behavior in the Y-maze test. The Y-maze test was carried out as described previously [11,12], on the 22nd–24th days. The modified fear conditioning test was performed on the 25th–30th days.

### 2.4 Modified Morris water maze test

Mice were placed in a water bath and trained to escape onto the platform in the pool for three consecutive days (11th–13th days; 18 male mice per each group) for the forward training session. The pool was 100 cm in diameter with opaque water at 22°C. The top surface of the hidden platform (10 cm in diameter) was placed 1 cm below the water surface. The time spent to find and escape onto the hidden platform was measured. The first trial was started at 9:30 am and three trial sessions per day were performed with an interval of about 30 min. In each training session, mice were allowed to swim until they found the platform or until 60 s had elapsed. Mice that failed to find the platform were guided to the platform and then allowed to remain there for 30 s. The platform was moved to a position opposite to that in the training period, and the reversal training was monitored for 2 days (17th and 18th days). The behavior of mice was recorded with a CCD camera (O’Hara & Co., Tokyo, Japan).

### 2.5 Fear conditioning test

This test consisted of two sessions: the conditioning and contextual sessions. During the conditioning session, the mice were placed in a chamber. They were supplied with a conditioned auditory stimulus and an unconditioned electric foot shock for several times. Freezing behavior emerges as fear to the insult. The freezing time during the conditioning session represents the levels of acquisition of fear-conditioned memory.

The conditioning chamber (length, 14 cm; width, 16 cm; height, 12 cm; O’Hara & Co., Tokyo) with a stainless-steel grid floor was installed in a soundproof box (200 lx, 55 dB white noise as a background noise). During the conditioning session, the mice were placed in the conditioning chamber and allowed to explore freely for 2 min. A tone of 65 dB white noise was sounded as the conditioned stimulus for 30 s, followed by a 2 s mild electric foot-shock (0.3 mA) as the unconditioned stimulus (a tone-shock pair). Two more tone-shock pairs were given at 2 min intervals, and their behaviors were monitored for 8 min (a conditioning session; Figure S2). Twenty-four hours after the conditioning session, the mice were placed again in the conditioning chamber and monitored for 5 min as a contextual session (Figure S2). During this session, they were exposed to the background noise without the unconditioned or conditioned stimulus. Photographs were taken every half second using a CCD camera. Freezing movements were automatically judged to occur by a recording and analyzing software (O’Hara & Co.), based on the movements of a mouse in two consecutive pictures.

### 2.6 Y-maze test

Briefly, the Y-maze consisted of three intersecting arms made of black acrylic panels. Each arm was 40 cm long, with a trapezoidal cross section consisting of a 10 cm top, a 3 cm bottom, and a 12 cm height. Each mouse was placed at the end of one arm and allowed to explore the Y-maze freely for 8 min, and the series of arm entries were recorded to evaluate short-term spatial working memory based on spontanous alteration (SA) behavior. The mice were considered to have chosen an arm when their entire torsos had entered the arm completely. SA behavior is based on the behavior of animals in preferring to choose newer locations. When the mice chose a third arm different from the previous two among the three consecutive arm choices in the Y-maze, the mice were considered to have carried out actual alternation. SA (%) was calculated as the percentage of actual alternations out of the maximum alternations that were defined as the total number of arm entries minus two. For example, if a mouse made 10 entries in an order such as arm A → B → C → B → A → B → C → B → A → C, the actual and maximum alternations would be 5 and 8, respectively, and alternation behavior would be 62.5%. The arm entries were also counted.

### 2.7 Inclusion and exclusion criteria

To exclude the effect of the sexual cycle on the results, only male mice were used for the experiments. If the mice did not move at all, they were excluded from analysis in the Morris water maze test. During the Y-maze test, if the number of the arm entries of a mouse was less than six, it was excluded from the analysis.

### 2.8 Preparation of interstitial fluid (ISF)-containing brain samples after the subcutaneous injection of CLSPCOL to mice

Some APP/PS1 mice aged 16 months received subcutaneous injection of 5 nanomol of CLSPCOL or saline every day for 14 days. Some Jcl:ICR mice aged 8 weeks received single subcutaneous injection of 5 nanomol of CLSPCOL. At times after the last injection of CLSPCOL, they were anesthetized with isoflurane (Wako Pure Chemicals, Tokyo, Japan). Blood was then aspirated from hearts and centrifuged at 4,000×*g* for 10 min at 4°C. The vascular space of the brain was washed free of blood by the perfusing 20 mL of ice-cold lactated-Ringer’s solution (Otsuka Pharmaceutical, Tokyo, Japan) through the left ventricle of the heart. Subsequently, the mice were decapitated and the brains were removed. The whole brain was once washed with the lactated-Ringer’s solution, to clear the contamination of CSF. The cortex was then separated by homogenizing with two-fold weight of saline. After the homogenate was centrifuged at 4,000×*g* for 10 min at 4°C, the supernatant was collected as the three-times diluted ISF-containing brain sample. The ISF-containing samples and sera were subjected to ELISA analysis.

## 3 ELISA

The single-step ELISA system for mouse CLSPCOL was generated as follows. Ninety-six-well plates (ELISA Plate H, cat. no.: MS-8896FZ, Sumitomo Bakelite, Tokyo, Japan) were filled with 100 μL of 1 mM streptavidin, purchased from New England Biolabs (cat. no.: N7021, Ipswich, MA), in PBS and incubated overnight. After blocking with PVDF Blocking Reagent (TOYOBO) for 1 h at 25°C and washing, the 96-well plates were filled with 100 μL of PBS containing 25 μg/mL of the biotin-conjugated mCLSP antibody, diluted ISF-containing samples, and peroxidase-conjugated mouse adiponectin antibody and incubated for 1 h at 25°C with shaking at 100 rpm. To make a standard curve or line, 100 μl of stepwise-increasing concentrations of mCLSPCOL and recombinant mouse CLSP-1 in PBS were filled in place of ISF-containing samples. After washing five times with 300 μl of the wash buffer, each well was filled with R&D TMB Substrate solution (cat. no.: DY999, R&D Systems), and the plates were incubated for 10 min at room temperature. The reaction was stopped by the addition of 50 μl of 2N H_2_SO_4_. Absorbance at 450 nm was measured using Wallac ARVO^TM^ X5 (Perkin Elmer).

### 3.1 Statistical analysis

All data were analyzed using Prism8 for Mac OSX (GraphPad, San Diego, USA). The data are shown as mean ± standard error of the mean (SEM). The statistical analysis was performed using two-way ANOVA, followed by *post hoc* Tukey’s multiple comparison tests for water maze and fear conditioning tests. For analysis of the Y-maze test non-parametric Kruskal–Wallis test, followed by Dunn analysis as a *post-hoc* test, was used. If the *p*-value was less than 0.05, it was considered statistically significant.

## 4 Results

### 4.1 CLSPCOL injection corrects the impaired fear-conditioned memory

**Figure 1:**
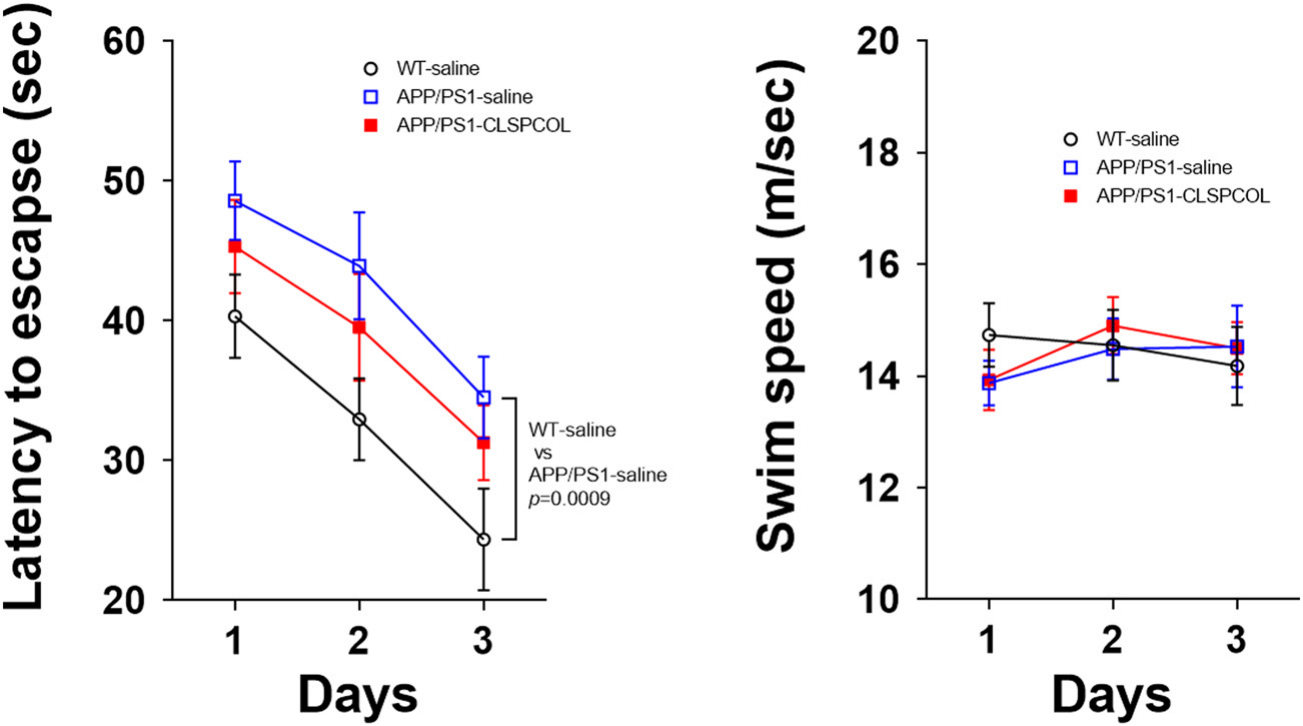
A modified Morris water maze test indicated that CLSPCOL tended to alleviate the deterioration of memory impairment in APP/PS1 mice at an age of 12 months. Modified Morris water maze trial tests were performed from the 11th to 13th days (forward test) with three trials per day. Mean ± SEM of the latency to escape (time spent to find and escape to the hidden platform) for each group on separate days is shown. The result of two-way ANOVA analysis of three-day experiment is as follows. Interaction [F (4, 153) = 0.05437, *P* = 0.9944]. Row [F (2, 153) = 15.55, *P* < 0.0001]. Column [F (2, 153) = 6.993, *P* = 0.0012]. The *post hoc* Tukey analysis revealed that *p* was statistically significant (0.0009) for the comparison of the WT-saline and APP/PS1-saline groups. However, *p* was insignificant (0.3581 or 0.0551) for the comparisons between the APP/PS1-saline and APP/PS1-CLSPCOL groups or between the WT-saline and APP/PS1-CLSPCOL groups. N is 18 for all groups. WT-saline group: inclusion (18/18,100%), exclusion (0/18, 0%). APP/PS1-saline group: inclusion (18/18, 100%), exclusion (0/18, 0%). APP/PS1-CLSPCOL group: inclusion (18/18, 100%), exclusion (0/18, 0%).

Using APP/PS1 mice aged 12 months, we first performed the modified Morris water maze test, according to the previous report [7]. Memory performance was already impaired in the APP/PS1 mice at this age (Figure 1 and Figure S3). Although the treatment of CLSPCOL showed the inclination to improve the memory performance, it did not improve it in a statistically significant fashion. We then performed the Y-maze test. The results indicated that the short-term working memory did not deteriorate in the APP/PS1 mice significantly (Figure S4). Consequently, we were unable to test the effect of the CLSPCOL treatment on the short-term working memory, although it tended to improve the memory performance in APP/PS1 mice (Figure S4).

**Figure 2:**
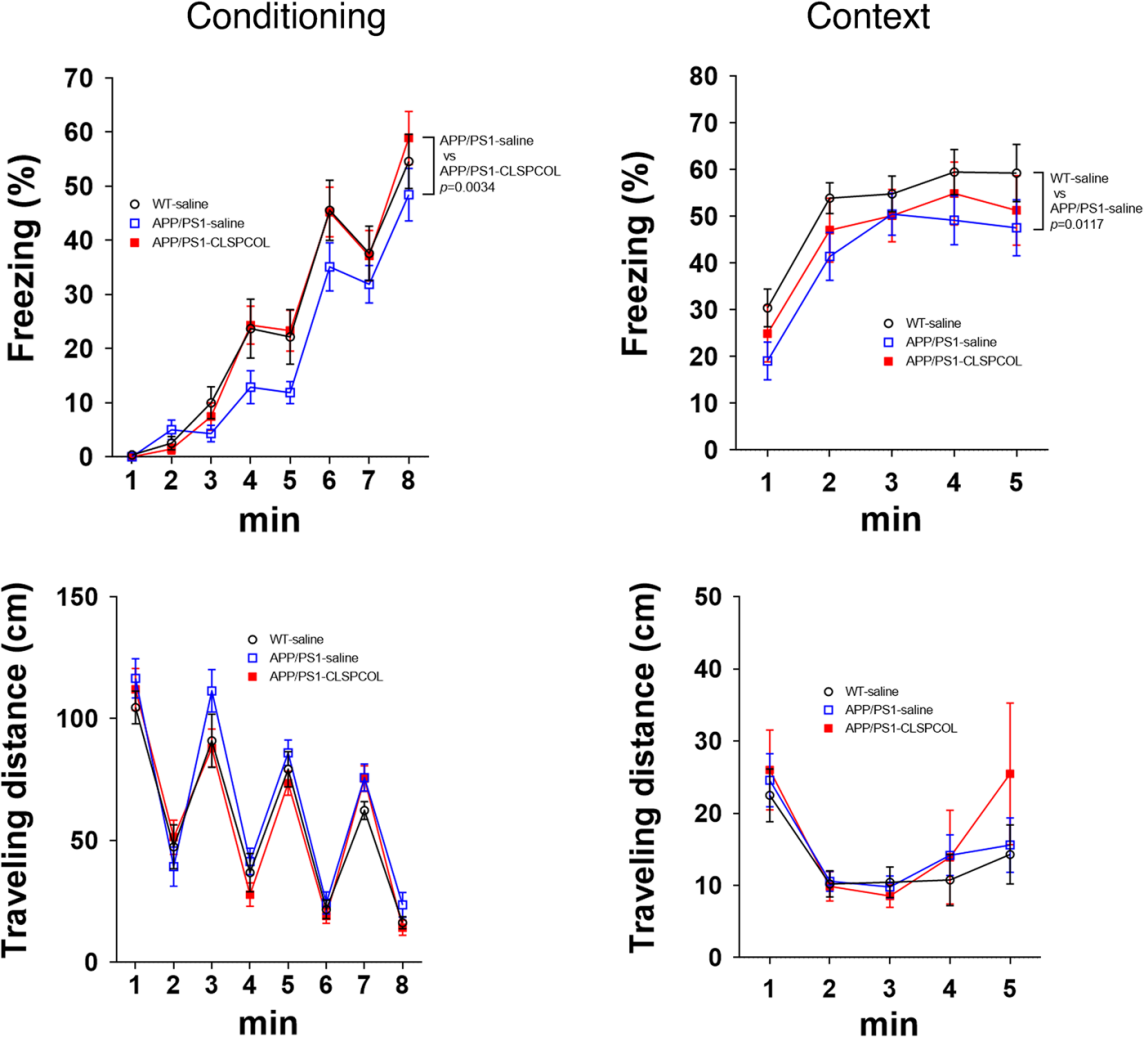
CLSPCOL rescued the deterioration of fear-conditioned memory. The modified fear conditioning test was performed from day 25 to day 30. Five or six mice per each treatment group were tested for two consecutive days. A total of 17–18 mice per each group were tested throughout the study period. The percentage of freezing movements per total movement for one minute was monitored. The freezing percentage for the first minute represents the percentage of freezing movements that were observed during the 0–60 second. The result of two-way ANOVA analysis of freezing percentage during conditioning session was as follows. Interaction [F (14, 400) = 0.7352, *P* = 0.7389]. Row [F (7, 400) = 84.12, *P* < 0.0001]. Column [F (2, 400) = 6.837, *P* = 0.012]. The *post hoc* Tukey analysis revealed that p was 0.0046, 0.0034, and 0.9955 for the comparisons between the WT-saline and APP/PS1-saline groups, the APP/PS1- saline and APP/PS1-CLSPCOL groups, and the WT-saline and APP/PS1-CLSPCOL groups, respectively. Two-way ANOVA analysis of freezing percentage during 5-min context session was as follows. Interaction [F (8, 250) = 0.1095, *P* = 0.9989]. Row [F (4, 250) = 15.12, *P* < 0.0001]. Column [F (2, 250) = 4.232, *P* = 0.0156]. The *post hoc* Tukey analysis revealed that *p* was 0.0117, 0.4629, and 0.1969 for the comparisons between the WT-saline and APP/PS1-saline groups, the APP/PS1-saline and APP/PS1- CLSPCOL groups, and WT-saline and APP/PS1-CLSPCOL groups, respectively. Two-way ANOVA analysis of traveling distance during the conditioning session was as follows. Interaction [F (14, 400) = 0.9203, *P* = 0.5368]. Row [F (7, 400) = 86.33, *P* < 0.0001]. Column [F (2, 400) = 3.258, *P* = 0.0395]. The *post hoc* Tukey analysis revealed that *p* was 0.0628, 0.0758, and 0.9965 for the comparisons between the WT-saline and APP/PS1-saline groups, the APP/PS1-saline and APP/PS1-CLSPCOL groups, and WT saline and APP/PS1-CLSPCOL groups, respectively. Two-way ANOVA analysis of traveling distance during the context session is as follows. Interaction [F (8, 250) = 0.4589, *P* = 0.8841]. Row [F (4, 250) = 6.434, *P* < 0.0001]. Column [F (2, 250) = 0.6892, *P* = 0.5029]. *N* = 17 for APP/PS1-CLSPCOL groups and *N* = 18 for the other two groups. One mouse belonging to the APP/PS1-saline group was excluded from the test because it had died before the start of the test. WT-saline group: inclusion (18/18, 100%), exclusion (0/18, 0%). APP/PS1-saline group: inclusion (17/17, 100%), exclusion (0/17, 0%). APP/PS1-CLSPCOL group: inclusion (18/18, 100%), exclusion (0/18, 0%).

We next examined the effect of the CLSPCOL treatment on the fear-conditioned memory, using a modified fear conditioning test. During the contextual session, the fear memory is recalled based on the conditioning environment. The APP/PS1 mice at this age had impaired fear-conditioned memory during both the conditioning and contextual sessions (Figure 2). The CLSPCOL treatment corrected the acquisition of fear-conditioned memory during the conditioning stage almost completely. Although it showed the inclination to correct the impairment of memory recalling during the contextual session, it did not correct it in a statistically significant manner (Figure 2).

### 4.2 A single subcutaneous injection of CLSPCOL rescues scopolamine-induced short-term working memory

**Figure 3:**
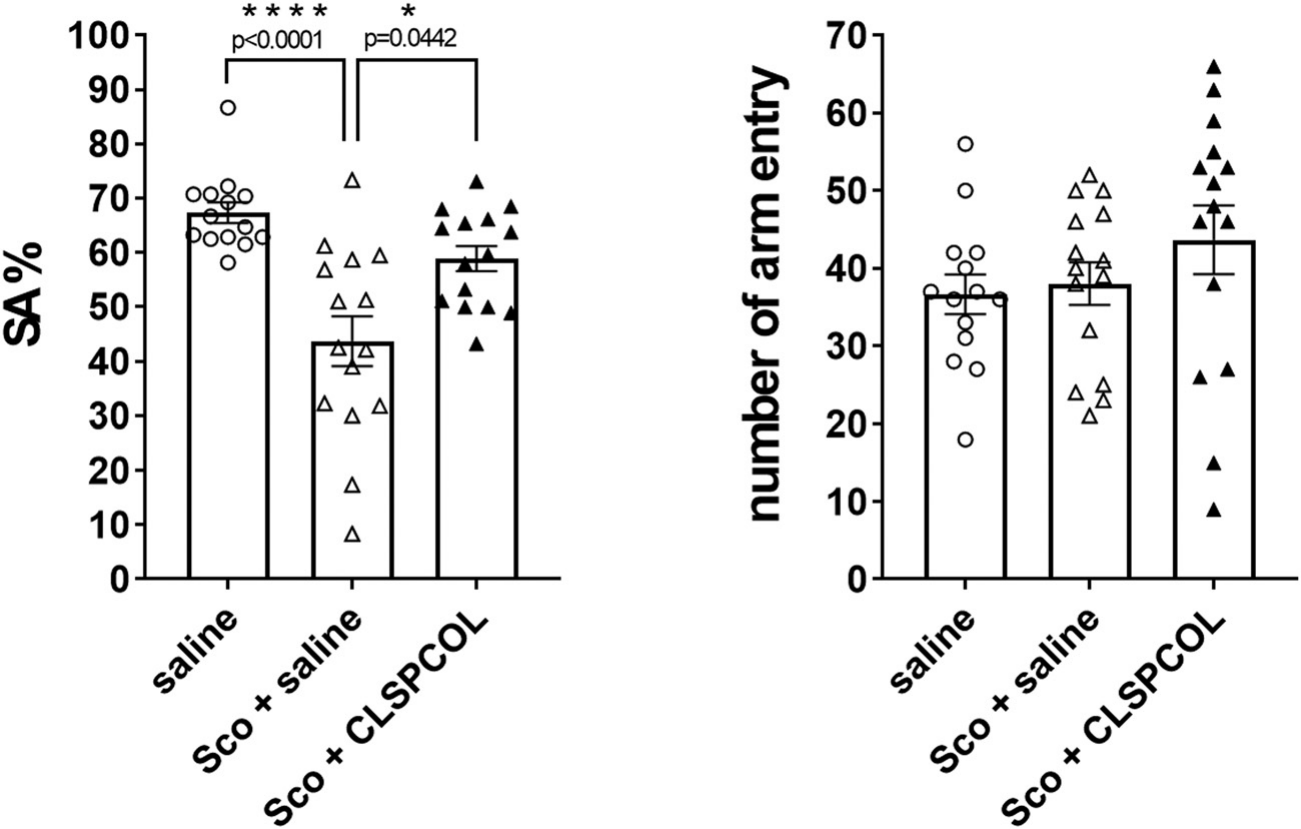
CLSPCOL relieved scopolamine-induced memory impairment. Jcl:ICR mice aged 10 weeks were subjected to the Y-maze test at one hour after the subcutaneous injection of scopolamine (indicated Sco) with/without CLSPCOL. The raw data and mean ± SEM of the spontaneous alternation (SA) percentage for each group (*N* = 14–15 for each group) are shown. Statistical analysis was performed using non parametric Kruskal–Wallis test, followed by Dunn analysis as a *post-hoc* test. Kruskal–Wallis test indicated that p was 0.0002 and 0.1676 for SA% and arm entry. The *post-hoc* Dunn analysis of SA % indicated that p was less than 0.0001 and 0.0442 for the comparisons between the saline and Sco + saline groups, and the comparison between the saline and Sco + CLSPCOL groups, respectively. The arm entries were counted as a locomotion index in the right panel. One mouse belonging to the saline group was excluded from analysis because the arm entry of the mouse was less than 6. saline: inclusion (14/15, 93.33%), exclusion (1/15, 6.67%). Sco + saline: inclusion (15/15, 100%), exclusion (0/15, 0%). Sco + CLSPCOL: inclusion (15/15, 100%), exclusion (0/15, 0%).

Scopolamine is an M2 muscarinic acetylcholine receptor antagonist. A single injection of scopolamine causes memory impairment in mice by disturbing cholinergic neurosignals. Previous studies showed that an intracerebroventricular or intraperitoneal injection of humanin or CLSP rescued scopolamine-induced memory impairment [11,12]. It is likely that CLSP antagonizes scopolamine by potentiating cholinergic neurotransmission via the htHNR [12,13]. In the current study, we found that a single subcutaneous injection of 5 nanomol of CLSPCOL suppressed scopolamine-induced impairment of short-term working memory, using the Y-maze test (Figure 3).

### 4.3 Measurements of CLSPCOL concentrations in ISFs and sera

**Figure 4:**
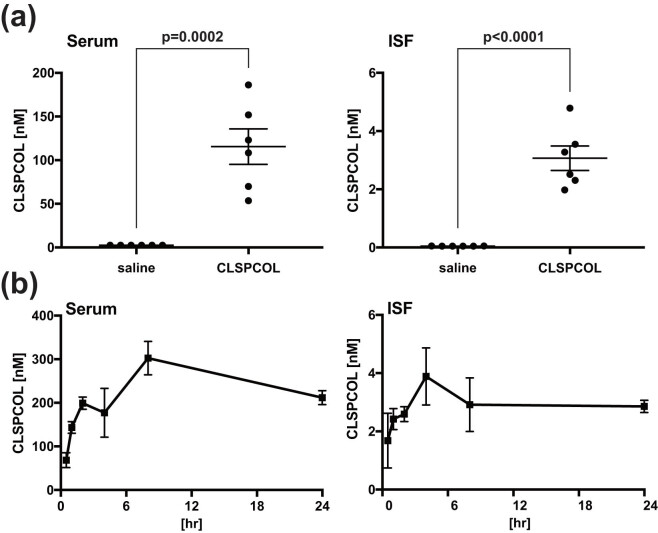
CLSPCOL concentrations were enough. (a) At 23–24 hr after the last subcutaneous injection of 5 nanomol of CLSPCOL or saline every day for 14 days, APP/PS1 mice aged 16 months were sacrificed for the preparation of ISFs and sera. CLSPCOL concentrations in ISFs and sera were determined using the ELISA analysis. The mean ± SEM of the concentrations are 3.067 ± 1.031 (*N* = 6). (b) At indicated time after the single subcutaneous injection of 5 nanomol of CLSPCOL, Jcl:ICR mice aged 8 weeks were sacrificed for the preparation of ISFs and sera. CLSPCOL concentrations in ISFs and sera of were determined using the ELISA analysis. The mean ± SEM of the concentrations are indicated (*N* = 3 for each point).

Using ELISA assay for CLSPCOL concentrations, we first measured the steady-state trough CLSPCOL concentrations in ISFs and sera of APP/PS1 mice aged 16 months. They received the subcutaneous injection of 5 nanomol of CLSPCOL every day for 14 days. The mean trough CLSPCOL concentration (3.067 nM) in ISFs of the mice was far more than the concentrations (0.1 nM) that are required for the complete inhibition of AD-related neuronal cell death *in vitro* (Figure 4a).

We also examined the time course of the ISF and serum concentrations of CLSPCOL after the single subcutaneous injection of 5 nanomol of CLSPCOL to Jcl:ICR mice aged 8 weeks. The mean CLSPCOL concentrations in ISFs of the mice within 24 h after the single subcutaneous injection were more than the concentrations (0.1 nM) that are required for the complete inhibition of AD-related neuronal cell death *in vitro* (Figure 4b).

## 5 Discussion

Using the modified Morris water maze test, we previously showed that CLSPCOL rescued memory impairment in the 16-month-old APP/PS1 mice (advanced-phase AD model) [7]. The difference in the memory function between the APP/PS1 and WT mice at this age was observed at relatively restricted points. Most so-called AD-modifying drugs such as anti-amyloid β monoclonal antibody named Aducanumab are hypothesized to be only effective against the early-phase AD. In the current study, we showed the efficacy of CLSPCOL using the same mice at a younger age (early-phase AD model), using another memory test, the modified fear conditioning test. Thus, the results of the previous study [7] and this study clearly suggest that CLSPCOL is the first disease-modifying drug that is effective against all-stage AD.

In contrast to the almost full recovery in the acquisition of fear-conditioned memory by the CLSPCOL therapy (conditioning session), only partial recovery was observed in the recalling of fear-conditioned memory by the CLSPCOL therapy (context session; Figure 2). The CLSPCOL-mediated recovery from memory impairment that was monitored using the water maze test was also partial (Figure 1). This is assumed to happen because neurons involved in each memory function were not necessarily identical and the speed of recovery from the synaptic loss by the CLSPCOL therapy was different among involved neurons. It is also possible that the response of the same-type neurons to the therapy is different among brain areas. Thus, it is anticipated that the recovery may become complete even for the recalling of the fear-conditioned memory and the memory monitored using the water maze test, if the CLSPCOL therapy will be delivered for sufficiently longer days.

AD-related neurotoxicity mainly occurs in the limbic system and the cerebral cortex. The acquisition of fear-conditioned memory is mediated by amygdala, whereas the recalling of the fear-conditioned contextual memory is mediated by both amygdala and hippocampus [14,15]. The results of the current study suggest that the CLSPCOL-mediated rescue of neurons occurs more rapidly in the amygdala than hippocampus.

We also showed that the one-shot CLSPCOL administration rescued the scopolamine-induced memory dysfunction (Figure 3). This result has been anticipated, because similar experiments previously indicated that the intraperitoneal injection of humanin or CLSP rescued the scopolamine-induced memory dysfunction, using the Y-maze test [11,12]. It is highly likely that the upregulation in the cholinergic nervous function by humanin or CLSP may occur by the stimulation of the htHNR-mediated intraneuronal signals [12,13].

Long-term treatment with donepezil, an acetylcholine esterase blocker, can rescue memory function in the AD model mice by potentiating cholinergic neurotransmission [16,17]. However, it did not correct the fear-conditioned memory impairment in the AD model mice [18]. In contrast, in the current study, CLSPCOL treatment rescued the deterioration in the fear conditioned memory in the early-phase AD model mice. Consequently, it could be concluded that the CLSPCOL-mediated recovery of the memory impairment in the early-phase AD model mice is not only mediated by the potentiation of the deteriorated cholinergic nervous system but also the direct suppression of AD-linked neurotoxicity.

The limitation of this study is that the result only indicates that the CLSPCOL therapy is effective against the synaptic loss of neurons linked to AD. It does not show that the CLSPCOL therapy is effective against the neuronal loss or death that occurs without exception and causes irreversible dementia in human AD. The used AD model mice do not suffer from apparent neuronal loss or death. Currently, there are no animal models that mimic neuronal loss or death in human AD cases. However, together with the previous results that CLSP or CLSPCOL suppresses AD-relevant death in cultured neuronal cells *in vitro* [2,3,7] and neuronal death is hypothesized to occur as a final neurotoxicity manifestation after neuronal dysfunction such as synaptic loss occurs, the results in the current study suggest that the CLSPCOL therapy will also be effective against neuronal death observed in human AD cases in addition to neuronal synaptic loss.

In summary, CLSPCOL likely serves as both a disease modifier via the suppression of AD-linked neurotoxicity and a symptomatic modifier via the potentiation of the neuronal cholinergic transmission for early- and advanced-phase AD. These results suggest that CLSPCOL is an ideal drug for the AD treatment.
